# The Functional Interplay between Ethylene, Hydrogen Sulfide, and Sulfur in Plant Heat Stress Tolerance

**DOI:** 10.3390/biom12050678

**Published:** 2022-05-08

**Authors:** Zebus Sehar, Harsha Gautam, Noushina Iqbal, Ameena Fatima Alvi, Badar Jahan, Mehar Fatma, Mohammed Albaqami, Nafees A. Khan

**Affiliations:** 1 Plant Physiology and Biochemistry Laboratory, Department of Botany, Aligarh Muslim University, Aligarh 202002, India; seharzebus5779@gmail.com (Z.S.); harshagautam99@gmail.com (H.G.); ameenafatimaalvi@gmail.com (A.F.A.); naziabadar.2014@gmail.com (B.J.); 2 Department of Botany, Jamia Hamdard, New Delhi 110062, India; naushina.iqbal@gmail.com; 3 Department of Biology, Faculty of Applied Science, Umm Al-Qura University, Makkah 21955, Saudi Arabia

**Keywords:** antioxidants, heat stress, mineral nutrients, post-translational changes, tolerance

## Abstract

Plants encounter several abiotic stresses, among which heat stress is gaining paramount attention because of the changing climatic conditions. Severe heat stress conspicuously reduces crop productivity through changes in metabolic processes and in growth and development. Ethylene and hydrogen sulfide (H_2_S) are signaling molecules involved in defense against heat stress through modulation of biomolecule synthesis, the antioxidant system, and post-translational modifications. Other compounds containing the essential mineral nutrient sulfur (S) also play pivotal roles in these defense mechanisms. As biosynthesis of ethylene and H_2_S is connected to the S-assimilation pathway, it is logical to consider the existence of a functional interplay between ethylene, H_2_S, and S in relation to heat stress tolerance. The present review focuses on the crosstalk between ethylene, H_2_S, and S to highlight their joint involvement in heat stress tolerance.

## 1. Introduction

### 1.1. Heat Stress: Impact and Consequences

In a field, plants are confronted with a variety of abiotic stresses; factors such as heat, drought, chilling, and salinity are all key constraints that impact crop yields in modern agriculture [1,2]. Of especial interest is the extreme seasonal heat induced by global warming, which exerts severe influence on crop growth and production around the world, aggravating food insecurity and malnutrition. In tropical and subtropical regions, an increase of 1 °C in seasonal temperature is expected to directly cause yield losses of 2.5% to 16% in staple crops [3]. According to the Intergovernmental Panel on Climate Change (IPCC), the greatest warming is regarded to occur in the period from the 19th to the 21st century. In the 21st century specifically, the average temperature of the Earth is anticipated to rise from 2 to 4.5 °C [4]. As the temperature has increased, the production of major crops has clearly been reduced around the world [5]; further temperature rise is expected to decrease yield more still. For example, forecasts based on the most conservative climate change projections suggest a minimum reduction of 4% to 10% in cereal production across South Asia [6]. Meanwhile, it is predicted that by 2050, rice production might drop by 8% and wheat production by 32% [7]. If temperatures rise by 3 to 4 °C, crop yields in Africa and Asia may be reduced by 15% to 35%, while those in the Middle East are likely to be reduced by 25% to 35% [8].

In conjunction with global warming, plant heat stress has become of considerable interest around the world, and the mechanisms of high-temperature injury and heat tolerance have attracted much attention [9,10,11,12]. Heat stress or shock is defined as a temporary increase in temperature of 10–15 °C over ambient conditions [13,14]. Whether temporary or persistent, high temperatures generate a variety of morpho-anatomical, physiological, and biochemical changes in plants, affecting their growth and development and consequently drastically lowering associated economic yield. Heat stress can affect a plant either directly or indirectly; protein aggregation, protein denaturation, and enhanced membrane fluidity are all examples of direct injury, whereas indirect injury can include inactivation of enzymes in chloroplasts and mitochondria, inhibition of protein synthesis, increased protein breakdown, and loss of membrane integrity. All of these changes cause cell injury or death within minutes, resulting in the catastrophic collapse of cellular organization [15,16,17]. At the process level, heat stress causes alterations in photosynthesis and respiration, resulting in a shorter life cycle and lower plant productivity [18]. For photosynthetic machinery in the chloroplast, the major sites of heat-induced damage have been identified as carbon metabolism in the stroma and chemical signaling in the thylakoid lamellae [19]. Both photosynthesis and the Calvin–Benson cycle enzymes, such as ribulose 1,5 bisphosphate carboxylase/oxygenase (rubisco) and rubisco activase, are extremely sensitive to increased temperature and become severely inhibited even at low levels of heat stress [20,21]. Meanwhile, the activity of carbon metabolism enzymes and the accumulation of starch and sucrose are adversely affected by elevated temperature through altered regulation of carbohydrate metabolism genes [22]. Heat-induced disruption to the photosynthetic apparatus and chlorophyll has also been connected to the production of reactive oxygen species (ROS) [23]. As an environmental factor, heat in its own right is further known to stimulate the generation of ROS, including superoxide anion radical (O_2_^−^) and hydrogen peroxide (H_2_O_2_); the resulting imbalance between ROS production and the available antioxidant defense leads to oxidative stress [24,25]. Heat stress thus causes irreversible damage to plants by influencing a wide range of cellular components and metabolic functions [26].

### 1.2. Physiological and Molecular Responses to Heat Stress

Heat stress adversely impacts physiological and biochemical responses and photosynthetic efficiency and reduces productivity [27] through disruption of thylakoid membranes and reduction in pigment system (PS)II activity [28]. Chloroplasts are sensitive to heat stress, and heat-stress-induced damage to chloroplasts leads to downregulation of important chloroplast components, inhibition of rubisco activity and decrease in photosynthetic efficiency with redox imbalance and possible cell death [29,30,31]. In addition, disruption of thylakoid membranes is a consequence of heat stress that reduces the rate of photosynthesis through a reduction in PSII activity. Plants exposed to heat stress show alteration in carbohydrate metabolism and disruption of membrane functions, with increased membrane permeability leading to loss of cellular electrolytes and decreased thermotolerance [32]. Water potential and relative water content are substantially decreased upon exposure to heat, reducing photosynthetic productivity [33]. The levels of soluble sugars and proteins are also altered during heat stress to regulate osmotic pressure within the cell [34]. Plants exposed to heat stress show excess ROS production that causes lipid peroxidation and membrane damage [35]. ROS may cause programmed cell death, but plants have developed a mechanism to detoxify ROS and endure tolerance through activation of the antioxidant system.

Heat stress is transcriptionally regulated by heat shock factors (HSFs) and heat shock proteins (HSPs) that interact to bring thermotolerance. In addition, heat-responsive genes are also regulated by transcription factors such as multiprotein bridging factor 1 C (MBF1 C), N acetylcysteine (NAC), WRKY, basic leucine zipper (bZIP), and MYB to induce tolerance [36]. Exposure to heat upregulates the NAC transcription factor which further regulates the expression of dehydration-responsive element-binding protein 2A (DREB2A) for heat tolerance [37].

Noncoding RNAs also play a significant role in response to heat stress. miR156 isoforms are induced by heat stress [36]. Similarly, miR398 is induced under heat stress, leading to the accumulation of ROS through downregulation of SODs and thus inducing HSFs and HSPs [36]. Various miRNAs, mRNAs, lncRNAs, and circRNAs are associated with the plant hormonal signal transduction pathway under heat stress in *Cucumis sativus* [37]. Ahmed et al. [38] reported that numerous ncRNAs were involved in the regulation of gene expression in response to stress in *Brassica*. A study by Yu et al. [39] found the involvement of long noncoding RNA in activating ethylene synthesis in apple [39] and tomato [40]. H_2_S pretreatment was found to regulate alkaline stress tolerance by regulating the expression of microRNAs through downregulation of mhp-miR408a expression and upregulation of mhp-miR477a and mhp-miR827 [31]. The crosslink of H_2_S with transcription factors, signal transduction, miRNAs, and epigenetic modifications has been studied to better understand the regulation of genes by H_2_S in plants [41].

Thus, we can predict that the noncoding RNAs that are important regulators in heat stress tolerance can be modified by H_2_S for stress tolerance and can probably modify ethylene synthesis. Therefore, a possibility exists that ethylene and H_2_S might interact for heat tolerance.

### 1.3. Regulation of Heat Stress by Phytohormones

In addition to the above effects, heat stress alters gene expression and transcript accumulation at the molecular level, causing stress-related proteins to be synthesized as a coping mechanism [42]. Phytohormones including ethylene, salicylic acid (SA), abscisic acid (ABA), brassinosteroids (BR), and jasmonate are responsible for integrating environmental and endogenous signals to control plant defense responses to several abiotic stresses, including heat stress [43]. In particular, ABA and SA help plants cope with the harmful consequences of heat stress by minimizing oxidative damage and maintaining photosynthesis. For example, in heat-exposed *Arabidopsis thaliana*, exogenous treatment with a low dose of methyl jasmonate has been found to maintain cell viability via controlling electrolyte leakage [44]. Meanwhile, BR has been shown to improve plant thermotolerance, namely by increasing the photosynthetic rate and elevating the expression of heat shock proteins (HSPs), which are implicated in the complex signal transduction network that allows plants to withstand heat stress. The BR signaling pathway also stimulates the expression of phytochrome-interacting factors and coordinates plant architectural modifications. In maize subjected to heat stress, application of ABA (100 µM) stimulates the expression of *sHSP17.2*, *sHSP17.4*, and *sHSP26*, as well as the activity of antioxidant enzymes, resulting in decreased cellular ROS levels [45]. Overall, most hormones ultimately improve plant heat tolerance through modulating ROS homeostasis, namely increasing antioxidant synthesis and thus the scavenging of ROS [43].

Heat stress also activates pathways for phytohormone biosynthesis, thereby resulting in greater hormone accumulation. In particular, Poór et al. [46] found that heat stress activates a number of ethylene biosynthesis and signaling genes; ethylene-mediated signaling in turn regulates the expression of HSPs. In addition to its direct role in heat stress tolerance, ethylene regulates the metabolism of ROS and reactive nitrogen species (RNS) via modulating osmoprotectants and the antioxidant defense system. In addition, ethylene has been shown to play a significant role in tomato pollen thermotolerance [47]. Finally, a study by Huang et al. [48] determined the ethylene response factor EIN3-ERF95/ERF97-HSFA2 transcriptional cascade to be key in heat stress response and established a connection between ethylene and its downstream regulatory factors in basal thermotolerance of plants. Ultimately, exogenous application of ethylene prior to or concurrent with heat stress has been found to considerably reduce heat-induced damage and improve plant thermotolerance, indicating a central role for ethylene in heat stress response. As such, there is considerable promise in targeting ethylene biosynthesis and signaling pathways with the aim of improving plant heat tolerance.

### 1.4. Sulfur-Containing Compounds in Heat Stress Tolerance

As a nutrient, sulfur (S) is a fundamental necessity for optimal plant growth and development and is required throughout the life cycle, from vegetative development through harvesting. It is a key component of plant proteins and contributes to fundamental processes such as electron transport, structure, and regulation [49,50]. In addition, while phytohormones regulate S metabolism, some require S or its derivatives for their biosynthesis [49]. Sulfur assimilates have also been reported to function as signaling agents for intracellular communication; in particular, thiol-containing sulfur metabolites including cysteine (Cys), methionine (Met), and glutathione (GSH) are well-known modulators of environmental responses, and sulfur compounds are required for the formation of effective defense mechanisms in response to a variety of stresses [51,52,53]. Accordingly, S status has a significant impact on the ability of plants to combat stressful conditions, and sulfur management is an important issue in crop plant nutrition. Studies aiming to mitigate heat stress in plants have primarily focused on introducing technologies to enhance crop performance; S in particular has attracted attention in this area due to its critical role in stress acclimation. For example, foliar treatment with sulfur has been shown to help tomato plants cope with heat stress and improve their physiological responses and growth. Sulfur addition has been found to alleviate oxidative damage induced by heat through an increase in ascorbate and glutathione content that lowers H_2_O_2_, MDA, and electrolyte leakage [54].

Sulfur is an important constituent of vitamins thiamine/vitamin B1 and biotin. The role of thiamine in heat stress has been suggested by Wolak et al. [55]. Proteome profiling of *Populus euphratica* upon heat stress by Ferreira et al. [56] showed the role of pyruvate dehydrogenase, of which sulfur is a cofactor, in heat stress.

Methionine, the sulfur-containing amino acid, plays an important role in heat stress response [57]. Chloroplast heat-shock proteins containing methionine are involved in protecting PSII electron transport during heat stress [58]. Methionine and polyamine derivatives are reported to play important role in stress tolerance. Polyamines provide heat tolerance by increasing the expression of stress response genes [59]. Proline, another amino acid, also regulates high-temperature-induced dehydration and tolerance and is induced under heat stress [57]. GSH is a sulfur-containing metabolite that combats stress either by working as a nonenzymatic antioxidant or through interaction with various signaling molecules that are activated under stress [60]. These signaling molecules are known to regulate HSP70 for heat tolerance [61], and in response to salinity, they increased ethylene production by regulating 1-aminocyclopropane-1-carboxylate [62]. Their interaction with ABA and ethylene regulates stress-related genes in response to abiotic stress [63,64].

Thioredoxin is a sulfur-containing heat-resistant protein, and in *A. thaliana*, thioredoxin reductase type C helped the plant tolerate stress [65]. Glucosinolate, another sulfur-containing secondary metabolite, increases under heat stress and has a protective role under heat stress [66].

### 1.5. Sulfur and Hydrogen Sulfide in Heat Stress Tolerance and Their Interrelationship with Ethylene 

S assimilation leads to the synthesis of methionine, which acts as a precursor for ethylene through S-adenosyl methionine (SAM), and hydrogen sulfide (H_2_S) is also produced by plant cells as an intermediate of assimilatory sulfate reduction. In plastids, plants reduce activated sulfate to sulfite, which is further reduced by sulfite reductase to H_2_S. The incorporation of H_2_S into O-acetylserine (OAS) to form cysteine catalyzed by O-acetylserine thiol lyase (OASTL) is a reversible reaction where cysteine could be decomposed to H_2_S and OAS [67]. Cysteine is a precursor for methionine which eventually leads to ethylene through SAM. Thus, the tolerance mechanisms induced by sulfur, H_2_S, and ethylene under heat stress could probably be interlinked with each other.

Interestingly, the emerging gasotransmitter H_2_S has also been revealed to possess important roles in abiotic stress tolerance; moreover, it seems to generally act via modulating the action of ethylene [68,69] H_2_S has been demonstrated to weaken the effect of ethylene on banana ripening [70], and ethylene in conjunction with H_2_S has recently been found to potentially alleviate hexavalent chromium toxicity in two pulse crops [71]. Hydrogen sulfide has also been shown by Kaya et al. [72] to mitigate mineral deficiency, specifically iron deficiency, in strawberry plants. Interestingly, treatment with the phytohormone ABA improves tobacco heat tolerance by increasing endogenous H_2_S, achieved through boosting the activity of L-cysteine desulfhydrase. Thus, H_2_S also factors into ABA-mediated heat tolerance [73]. Hancock [74] highlighted that in its central role in the S cycle, plant-produced H_2_S may also have intracellular effects through the modification of thiols and antioxidants. However, the potential mechanisms by which the S-assimilation pathway might regulate ethylene and H_2_S biosynthesis under heat stress are as of yet unknown.

More broadly, abiotic stress tolerance is aided by the coordination of phytohormones and nutritional signals. That is, plant nutrients interact with phytohormones, signaling molecules, polyamines, and even other nutrients, and these interactions can produce derivatives that counteract abiotic-stress-induced adversity and enhance stress tolerance [75,76]. The phytohormones ethylene and SA are representative of such interactions, as they both regulate S metabolism and influence abiotic stress tolerance [77]. For example, ethylene enhances ATP-sulfurylase (ATP-S) activity and Cys and GSH content in salt-stressed plants, and the same was reported with the application of nitrogen (N) and S [78]. These observations demonstrate the common regulatory impacts of ethylene, N, and S upon components of the salinity stress response. In relation to plant heat tolerance, studies have mainly investigated phytohormones and plant nutrients separately. While their interaction has been explored in relation to other abiotic stresses, little research has been done on the interaction of phytohormones and nutrients in the context of heat stress. Importantly, as the synthesis of ethylene and H_2_S needs S as a backbone, it is postulated that there is connectivity between ethylene, H_2_S, and S in heat stress tolerance. The present review focuses on the functional interplay between ethylene, H_2_S, and S in heat stress tolerance. The present review first focuses on the linkage of ethylene and H_2_S with S. Then, the individual role of each of these in heat stress tolerance is discussed to work out their mechanism of action and to find a common interface among them. Lastly, crosstalk between ethylene and H_2_S for heat stress tolerance through the involvement of sulfur is discussed.

## 2. Ethylene and H_2_S Synthesis: Involvement of the Sulfur Assimilation Pathway

**Figure 1:**
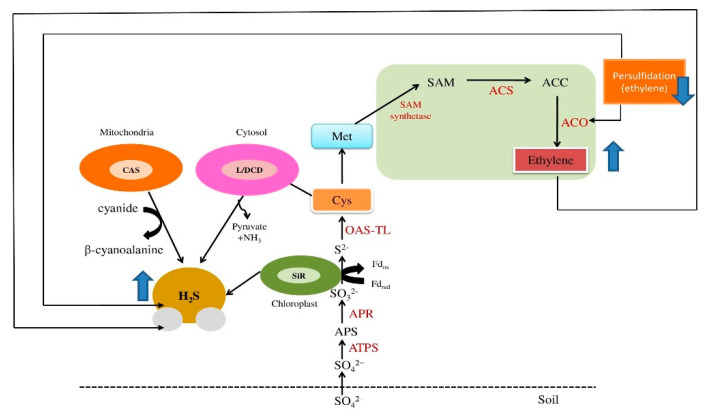
Schematic representation of the synthesis of H_2_S and ethylene and its association with the S-assimilation pathway. In plants, absorption of S occurs through uptake of SO_4_^2^^−^ from the soil by roots. The assimilatory pathway is activated to produce APS from SO_4_^2^^−^ under catalysis by ATP-S, which is in turn reduced to SO_3_^2−^ via APR. Afterward, SO_3_^2−^ is reduced to S^2−^, which is used to produce H_2_S via catalysis by chloroplast-localized sulfite reductase. The subsequent catalyzation of S^2−^ by OASTL yields Cys, which is the first stable compound in the S-assimilation pathway and the precursor for GSH and Met. Met is converted to SAM by SAM synthetase, from which ACC is synthesized by ACS, degradation of which by the ACO enzyme yields ethylene. Ethylene-induced H_2_S in turn regulates ethylene biosynthesis via the persulfidation of ACO. H_2_S can also be generated through degradation of Cys or through biosynthesis in mitochondria and cytosol by the enzymes CS, CAS, LCD, and DCD. Activity of LCD and DCD in the cytosol is accompanied by formation of pyruvate and NH_3_. ACC, 1-aminocyclopropane-1-carboxylic acid; ACO, ACC oxidase; ACS, ACC synthase; APR, APS reductase; APS, adenosine 5-phosphosulfate; ATP-S, ATP-sulfurylase; CAS, β-cyanoalanine synthase; Cys, cysteine; CS, cysteine synthase; DCD, D-cysteine desulfhydrase; GSH, glutathione reductase; LCD, L-cysteine desulfhydrase; NH_3_, ammonia; Met, methionine; S, sulfur; SAM, S-adenosyl methionine; SO_4_^2−^, sulfate; SO_3_^2−^, sulfite; S^2−^, sulfide; SiR, sulfide reductase; OASTL, O-acetylserine (thiol)-lyase. Blue arrows indicate upregulation and downregulation.

Sulfur is a crucial element required for all living organisms as an active component of amino acids (i.e., Cys and Met), vitamins, GSH, several group transfer coenzymes, and the thioredoxin system that fulfills vital functions in plant growth and development [79,80]. Of particular relevance to the present topic is that assimilation of S is directly or indirectly involved in the biosynthesis of H_2_S and ethylene (Figure 1). In plants, there are two routes for absorption of S: either sulfate (SO_4_^2−^) is taken up from the soil by roots, or sulfur oxides are absorbed from the atmosphere by leaves through the stomata [81,82]. However, the second mode mainly occurs when the soil is S-deficient; soil absorption is preferred. Once inside the roots, SO_4_^2−^ is first reduced and then incorporated into organic compounds [83]. Meanwhile, in leaf tissues, both assimilation and reduction occur as the enzymes involved in these processes are restricted to chloroplasts [84]. After entry of SO_4_^2−^ into chloroplasts, the assimilatory pathway is activated to produce adenosine 5-phosphosulfate (APS) through catalysis by ATP-sulfurylase (EC 2.7.7.4); APS is then in turn reduced to sulfite (SO_3_^2−^) via APS reductase (APR, EC 1.8.99.2) with GSH as the electron donor. Afterward, SO_3_^2−^ is reduced to sulfide (S^2−^) under catalysis by sulfite reductase (SiR, EC 1.8.7.1) [78,85]. The sulfide is then used to produce H_2_S, for which SiR is considered a major generating enzyme in the chloroplast [86,87]. Subsequently, H_2_S and O-acetylserine are catalyzed by O-acetylserine (thiol)-lyase (EC 2.5.1.47) to produce Cys, which is the first stable compound of S assimilation and the synthetic precursor for both GSH and Met [88]. It can also be degraded to generate H_2_S. Recent studies have revealed that in addition to SiR, a variety of enzymes in the mitochondria and cytosol contribute to the biosynthesis of H_2_S, such as cysteine synthase (CS), β-cyanoalanine synthase (CAS, EC 4.4.1.9), L-cysteine desulfhydrase (LCD, EC 4.4.1.28), and D-cysteine desulfhydrase (DCD, EC 4.4.1.15) [48,87]. In mitochondria, H_2_S can be produced by CAS in the course of cyanide detoxification during ethylene synthesis, which requires the release of S^2−^ through catabolism of Cys [89]. Importantly, accumulation of S^2−^ is lethal for plants, and so detoxification mechanisms regulate its formation during the assimilation of S and/or metabolism of cyanide. An imperative detoxification mechanism involves fixation of S^2−^ into Cys under severely elevated levels of S^2−^ [90]. In addition, H_2_S generation from Cys in the cytosol mainly occurs through the activity of LCD and DCD, which is accompanied by the formation of pyruvate and ammonia [91,92] (Figure 1).

The rate-limiting enzyme in S assimilation is APR, which controls the flow of inorganic S into Cys and thus likely controls endogenous H_2_S production [93], which occurs via catalysis by enzymes downstream of APR [94,95]. Notably, H_2_S has the capability to interrelate with thiol (-SH) groups that are present in peptides such as GSH, and also with proteins that modify them, namely those that convert Cys thiols (-SH) into persulfide thiols (-SSH), a reaction known as persulfidation [96,97]. Such oxidative post-translational modification of Cys residues represents a signaling mechanism and is involved in biosynthetic pathways that require the transfer of S, for example in producing S-containing bases in RNA, Fe-S clusters, thiamine, biotin, molybdopterin, and lipoic acid [86]. In addition, these modifications of Cys residues can act as a defensive mechanism in an oxidative stress environment.

Apart from its free state, H_2_S reacts with different biochemical molecules and establishes different bioavailable pools including stable, acid-labile, and bound sulfide forms [98]. Free sulfide exists as S^2−^, HS^−^, or H_2_S. Acid-labile sulfide is fundamental for iron–sulfur (Fe-S) complexes and persulfides, which assume a basic part in redox responses in cytoplasm and mitochondria. The critical pH below which H_2_S is released out of acid-labile sulfur-like Fe-S is 5.4 [99]. On the other hand, bound sulfane sulfur exists as a compound containing sulfur-bonded sulfur [100]. This incorporates compounds such as polysulfides, thiosulfate, polythionates, thiosulfonates bisorganyl-polysulfanes or monoarylthiosulfonates, and elemental sulfur. It may be said that H_2_S interconverts between gaseous and other complex storage compounds involving pH. Further, it has been reported that oxygen concentration and pH affect the stability of sulfide and derivatization within biological samples [101].

The other S-containing amino acid, Met, is synthesized in three steps from Cys and then is either incorporated into proteins or converted to S-adenosyl methionine (S-AdoMet (SAM)) by SAM synthetase (SAMS, EC 2.5.1.6) [80]. S-adenosyl methionine is the precursor of ethylene, biotin, polyamines, nicotinamide, and many other secondary metabolites [83]. Ethylene synthesis depends on SAM and likewise occurs in three steps; first, S-adenosyl methionine is formed from Met by the action of SAM synthetase, after which 1-aminocyclopropane-1-carboxylic acid (ACC) is synthesized by ACC synthase (ACS, EC 4.4.1.14). Subsequently, ethylene is produced from the degradation of ACC by ACC oxidase (ACO, EC 1.14.17.4) [80,102]. The latter two enzymes, ACS and ACO, are encoded by multigene families [102]. Notably, derivation of ethylene from Met is not the only link between S assimilation and ethylene biosynthesis; the transcription factor ethylene insensitive-like 3 (EIL3) regulates many S-deficiency-responsive genes [103,104], primarily through the key regulator sulfate limitation 1 (SLIM1). Thus, there is a significant link between S status and ethylene signaling.

## 3. The Crucial Roles of Ethylene, H_2_S, and S in Heat Stress Tolerance

### 3.1. Potential Role of Ethylene in Heat Stress Tolerance

Heat stress affects plants, bringing about changes at the molecular, biochemical, morphological, and physiological levels, including increased ethylene biosynthesis. For example, soybean plants exposed to high temperature (38 °C for 14 days) showed enhanced ethylene production in leaves and pods [105]. Similarly, the heat-susceptible wheat cultivar ‘Karl 92′ had higher ethylene production in flag leaves, embryos, and kernels than did the heat-tolerant cultivar ‘Halberd’ [106]. In rice, application of the ethylene precursor ACC enhanced seedling tolerance to heat stress and reduced levels of cell membrane oxidation and ion leakage [107]. In conjunction with these effects, transcript expression was upregulated for the ethylene biosynthesis genes ACC oxidase 1 (*ACO1*) and ACC oxidase 3 (*ACO3*), which encode enzymes that catalyze ACC into ethylene; also elevated were the associated signal transduction genes ethylene-insensitive (EIN) 2, EIN-like1, and EIN-like2 (*OsEIN2*, *OsEIL1*, and *OsEIL2*). Work by Larkindale and Knight [108] further suggests that ethylene contributes to protecting *Arabidopsis* from heat-induced oxidative damage. Similarly, ethylene has been shown to play a favorable role in the activation of antioxidant enzyme activities and the reduction in ROS levels in plants under severe temperature stress. For instance, 10 µM ACC in *Oryza sativa* seedlings exposed to 45 °C for four days activated the antioxidant enzymes catalase, ascorbate peroxidase, and total peroxidase, which were positively linked with increased tolerance [107]. Likewise, exogenous application of ethephon (an ethylene-releasing compound) to tomato plants prior to heat stress (50 °C for 2 h) upregulated protective mechanisms against oxidative stress, specifically those involved in cellular redox balance (including increased abundance of protein disulfide isomerase, glutathione-disulfide reductase, and glutaredoxin) [109]. Pretreating creeping bent-grass plants with 100 µmol/L ACC similarly imparted thermotolerance by maintaining higher ascorbate peroxidase, catalase, and peroxidase activity [110]. Thus, ethylene aids in ROS detoxification by inducing the antioxidant defense system during heat stress, allowing for heat stress adaptation.

Participation of ethylene in heat stress response and thermotolerance is further supported by results showing that treatment with an ethylene inhibitor negatively affects these changes, whereas using ethylene precursors promotes them. As an example, tomato plants of the ethylene-insensitive mutant never ripe (Nr), which are defective in an ethylene response sensor (ERS)-like ethylene receptor, exhibited higher heat-stress sensitivity. Likewise, a heat-susceptible wheat cultivar has been found to feature reduced ethylene production with increased kernel abortion and reduced kernel weight [106]. Meanwhile, pretreating wild-type tomato plants with an ethylene releaser in advance of heat stress improves pollen quality and increases the number of germinating pollen grains; conversely, pretreatment with an ethylene biosynthesis inhibitor reduces the number of germinating grains [47]. Similarly, priming lettuce seeds with 10 µM ACC added into KH_2_PO_4_ solution improves seed germination and performance under high temperature [111], and the application of exogenous ethylene precursors in artichoke promotes seed thermodormancy and reduces early root inhibition under heat stress [112]. More generally, pretreatment of a cool-season grass with ACC prior to heat exposure was found to boost thermotolerance, as evidenced by grass quality and leaf photosynthetic rates [110]. In addition, proteomics analysis revealed that pretreatment of lettuce with ethephon prior to heat stress causes the heat-stress pollen proteome to more closely resemble that associated with nonstressful conditions, specifically exhibiting a greater abundance of proteins involved in protein synthesis, degradation, the tricarboxylic acid cycle, and RNA regulation [109]. However, while a positive role of ethylene in heat tolerance is widely accepted, contradictory findings have been reported. For example, the heat-stress-induced elevation of ethylene production triggers premature leaf senescence in soybean, whereas an ethylene production inhibitor (1-methylcycloprpene (1-MCP)) reduces or postpones premature leaf senescence [105]. Ethylene has also been linked to a yield penalty in heat-stressed wheat crops, in which context the application of compounds that serve as ethylene biosynthesis antagonists results in a considerable increase in grain output [106,113,114].

Moreover, under heat stress, protection of photosynthesis was associated with the physiological and biochemical mechanisms of SA, such as increased synthesis of proline and N assimilation and suppression of stress ethylene production [24]. It has also been reported that ethylene production was increased under heat stress and caused a decrease in grain yield [115]. Indeed, metabolite profiling during heat stress showed an accumulation of organic acids, sugar alcohols, and sucrose after treatment with AVG, an ethylene biosynthesis inhibitor, while glucose and fructose levels were greatly reduced [116], suggesting that differential accumulation of metabolites involved in osmoregulation and antioxidant metabolism induces tolerance to heat stress in the absence of ethylene [116]. Some selected studies on the role of ethylene in heat stress tolerance are shown in Table 1.

A key element of the ethylene signaling and response pathway is ethylene response factors (ERFs), which belong to the well-known APETALA2/ethylene-responsive element-binding protein (AP2/EREBP) family [117]. Plants exposed to heat stress show enhanced expression of ERFs, and *ERF1*-overexpressing *Arabidopsis* plants exhibit greater tolerance to heat shock treatment than do control plants [118]. Huang et al. [119] discovered that ERFs regulate several heat-responsive genes in a heat-inducible way. A plant’s ability to respond to heat stress is mediated by heat shock transcription factors (HSFs), which quickly become activated under heat stress and connect to the promoter elements of genes encoding HSPs to boost their transcription [120]. HSPs then act as molecular chaperones, preventing cellular proteins from aggregating and denaturing while also assisting in the refolding of heat-damaged proteins [121]. In an *ERF1*-overexpression *Arabidopsis* line, heat shock treatment increased transcript levels of *HsfA3* and *HSP70*; mechanistically, ERF1 bound to the GCC box elements of *HsfA3* and *HSP70* promoters and upregulated their expression to improve thermotolerance [118]. A recent study conducted by Wu and Yang [107] further revealed that ethylene signaling is involved in the complex regulation of HSF genes during heat stress; in particular, combined heat and ethylene precursor treatment in rice seedlings resulted in higher expression of the *HsfA1a* and *A2a*, *c*, *d*, *e*, and *f* genes than did heat stress alone.

### 3.2. Role of H_2_S in Heat Stress Tolerance

Long thought to be a phytotoxin, hydrogen sulfide is now recognized as a cell-signaling molecule involved in higher plant growth, development, and stress tolerance. It is actively associated with the plant defense system under severe conditions. In vitro, Li et al. [122] found that pretreatment of suspension-cultured tobacco cells with the H_2_S donor sodium hydrosulfide (NaHS) could improve heat tolerance and that such acquisition of tolerance requires entry of extracellular Ca^2+^ across the plasma membrane, as well as mediation by intracellular CaM. Likewise, pretreatment of wheat seedlings with NaHS has a positive dose-dependent effect on heat tolerance that is specifically related to H_2_S [123]. Contrarily, Zhang et al. [124] showed contradictory findings on the role of exogenous H_2_S in heat stress tolerance. A high concentration of H_2_S inhibited primary root growth via the ROS-MPK6-NO signaling pathway. Exogenous H_2_S repressed the distribution of auxin and reduced the meristematic cell division potential in root tips, and NO was involved in this process. It has been reported that the low concentrations of H_2_S improved the tolerance of plants to abiotic and biotic stress, but high concentrations induced toxicity in plants’ growth [125]. However, Li et al. [126] found that pretreatment of maize seedlings with NaHS increased seed germination and seedling survival under heat stress and reduced root electrolyte leakage, tissue vitality, and the build-up of malondialdehyde (MDA) in coleoptiles. Such pretreatment also increased the activity of Δ1-pyrroline-5-carboxylate synthetase (P5CS) and decreased that of proline dehydrogenase (ProDH), resulting in a build-up of endogenous proline. Christou et al. [127] likewise found that pretreating strawberry (*Fragaria* × *ananassa* ‘Camarosa’) roots with NaHS effectively alleviated heat-associated decreases in leaf chlorophyll fluorescence, stomatal conductance, and relative leaf water content, as well as increases in ion leakage and MDA accumulation. Additionally, endogenous H_2_S improved mechanical stability and physiological functions by increasing the uptake of key nutrient elements, i.e., calcium and potassium, in strawberry plants [128].

Ultimately, the beneficial effect of H_2_S is attributed to its ability to activate the antioxidant system, thus reducing the oxidative damage associated with stress conditions. During high temperature stress, the activities of superoxide dismutase (SOD), catalase (CAT), and ascorbate peroxidase (APX) in NaHS-pretreated seedlings are increased compared to those of stressed non-pretreated plants, maintaining ROS homeostasis [123]. Min et al. [129] likewise suggested that exogenous NaHS treatment in wheat seedlings alleviates oxidative damage and increases heat tolerance by modulating antioxidant enzyme activity and gene expression under heat stress. Furthermore, at eight hours after heat stress exposure, strawberry plants whose roots were pretreated with NaHS were able to conserve ascorbate–glutathione (AsA-GSH) equilibrium, demonstrated by lower AsA and GSH pool redox disturbances and increased transcription of AsA and GSH biosynthesis enzymes; pretreatment also enhanced gene expression of antioxidant enzymes (*cAPX*, *CAT*, *MnSOD*, *GR*), heat shock proteins (*HSP70*, *HSP80*, *HSP90*), and aquaporins (*PIP*) [130]. These findings imply that H_2_S pretreatment activates a coordinated network of pathways related to heat shock defense, including antioxidant defense, at the transcriptional level, protecting plants from heat-stress-induced damage on a systemic level [131]. Interestingly, SA-induced heat tolerance in maize was found to be promoted by NaHS and blocked by H_2_S biosynthetic inhibitors or scavengers [132]. Therefore, H_2_S functions downstream of SA. Taken together, these findings suggest that heat tolerance in plants can be improved by NaHS pretreatment and that acquisition of NaHS-mediated heat tolerance may necessitate synergistic effects of the antioxidant system, the calcium messenger system, and heat shock proteins. Table 2 shows some selected studies on the role of H_2_S in heat stress tolerance.

### 3.3. Potential Role of Sulfur/S Compounds in Heat Stress Tolerance

Photosynthesis is more heat-sensitive than dark respiration and ceases before respiration is impaired on account of the damage induced by high temperatures [136]. High temperatures also induce a lack of transport that causes water shortage in plant tissues, resulting in mineral deficiency [137]. Mineral nutrition is key to regulating plant growth, metabolic functioning, and stress mitigation, and so it is of particular interest when it comes to developing new technologies and approaches for increasing crop performance under heat stress. Among all mineral nutrients, S is considered particularly key in the context of heat exposure [49]. Plants use S as a signaling agent to facilitate communication within the cellular environment [52], and they require thiol-containing biomolecules to establish defense mechanisms against various abiotic stressors [138]. For example, amino acids and metabolites containing S act to increase thermotolerance through interaction with a variety of biological substances, including plant growth regulators, enzymes, polyamines, and nutrients, and furthermore produce compounds that are essential in mechanisms for mitigating heat stress [49]. Sulfur and its derivatives are also essential for the activation of ROS-scavenging enzymes, which improve antioxidant defense in the face of abiotic stress [139]. In addition, sulfur is linked to secondary metabolism, abiotic and biotic stress regeneration, and photosynthetic oxygen generation. Mobin et al. [140] confirmed the involvement of sulfur in heat stress mitigation through significantly increasing photosynthetic and growth parameters, increasing antioxidant enzyme activities, and reducing oxidative stress biomarkers such as MDA and electrolyte leakage. The sulfur-containing defense compounds crucial for plant survival during biotic and abiotic stress response include elemental S, H_2_S, GSH, phytochelatins, S-rich proteins, and secondary metabolites, and the availability, demand, uptake, and assimilation of S are all critical factors in the formation of those compounds [50]. All told, many previous studies have emphasized the importance of S in plant stress defense. 

In addition to the above, Martins et al. [141] found that iron–sulfur glutaredoxin (GRXS17) is redox-modified and consequently activates holdase to protect plants from heat stress. It also alleviates the misfolding and aggregation of proteins induced by heat stress. Consequently, we believe that the Fe-S cluster enzyme GRXS17 is an important guardian that protects proteins from moderate heat stress, most likely via a redox-dependent chaperone function. Some selected studies on the role of S in heat stress tolerance are shown in Table 3.

## 4. Post-Translational Modification of Ethylene- and H_2_S-Associated Proteins under Heat Stress 

Post-translational modification (PTM) is a crucial step in determining the final functional fates of proteins. In post-translational modification, amino acid residues undergo covalent modifications such as glycosylation, phosphorylation, methylation, ADP-ribosylation, oxidation, and glycation; it can also involve proteolytic cleavage of the peptide backbone, nonenzymatic modifications such as deamidation and racemization, and spontaneous changes in protein conformation [147]. These modifications ensure the proper assembly and folding of proteins, which are then secreted or targeted to various compartments of the secretory system [148]. Post-translational modifications have also been found to perform significant roles in stress-exposed plants, for example helping to reduce crop damage [149]. Abiotic stresses in general exert significant impacts on plant proteomes, causing alterations in relative protein quantity, localization within the cell, post-transcriptional and -translational modifications, stability, interactions, and functions [150]. 

Of the various post-translational modifications in plants, SUMOylation has been revealed as a key player in responses to environmental stresses [149]. Studies conducted by Kurepa et al. [151] and Saracco et al. [152] using anti-SUMO antibodies discovered the crucial role of SUMOylation in defending against abiotic stresses such as heat, alcohol, ROS, and pathogenic toxicity. A positive role of SUMOylation in abiotic stress is also supported by various studies conducted on rice [153,154], tomato [155], and tobacco [156]. Enhanced SUMOylation of proteins under stress conditions such as higher temperature, drought, salinity, and increased ROS level further suggests a direct connection of this modification with tolerance response [151]. Mechanistically, a study in *Arabidopsis* indicated that protein modification mediated by SUMO1 and SUMO2 in the context of heat stress is directed by SIZ1 [157]. The direct relationship between SIZ1 and HSPs was explored by Zhang et al. [158] in heat-exposed tomato and revealed overexpression of SIZ1 followed by increased SUMO conjugation in such plants. In addition, an investigation of chromatin remodeling determined that protein SUMOylation is increased at promoters and enhancer sites during heat stress [159].

Furthermore, noncoding RNA is involved in the post-translational modification of ethylene and H_2_S. Noncoding RNA is the class of RNA regulating gene expression; however, these RNAs do not code for any functional proteins. microRNAs (miRNAs), small interfering RNAs (siRNAs), long noncoding RNAs (lncRNAs), and circular RNAs (circRNAs) are important noncoding RNAs involved in translational and post-translational gene modification [160,161]. Recent reports show a strong regulatory mechanism of such RNAs in overcoming abiotic stress responses in plants. In *Arabidopsis*, miR398 was rapidly induced in response to heat stress, followed by the downregulation of its target genes (CSD1, CSD2, and CCS). These are highly conserved genes involved in ROS scavenging [162]. Presences of the *miR398*-*CSD*/*CCS* pathway were also reported in plants like *Brassica rapa* and *Populus tomentosa* [163,164]. The association of miRNAs with phytohormones was also studied in several plants under heat stress. miR390, miR393, miR160, and many other miRNAs are involved in the auxin signaling pathway under heat stress. *AUXIN RESPONSE FACTOR17* (*ARF17*) and *ARF13* genes are the target sites for miR160. In the case of wheat, miR160 was downregulated while its other target *HSP70* was upregulated when exposed to heat [165]. On the other hand, miR159 negatively targets the *GAMYB* genes of GA [166]. Plant mutants overexpressing *TamiR159* are heat-sensitive since overexpression of miR159 leads to *GAMYB* downregulation during heat stress [167].

Small interfering RNAs (21–24 nucleotides long) catalyze dsRNA processing. In the case of wheat, the levels of siRNAs were downregulated by heat stress and upregulated by cold stress [168]. In *Arabidopsis*, restriction of the *ONSEN* gene is regulated by siRNA under heat stress [169]. Negative regulation of *HEAT-INDUCED TAS1 TARGET1* (*HTT1*) and *HTT2* was seen when miR173-cleaved ta-siRNA (*TAS1*) was overexpressed, leading to poor thermotolerance [161,170]. lncRNAs are 200 nt in length; they are classified as antisense lncRNAs or intronic lncRNAs based on their location [171]. Fifteen heat-responsive lncRNAs and 34 lncRNAs were identified in *Arabidopsis* and *B. rapa*, respectively [172]. Moreover, *SUCROSE SYNTHASE 4*, which is a heat-responsive gene, is the target site for lnc-173 under heat stress. Noncoding RNAs are the potent regulators of heat stress responses. Although genome-scale approaches have been used to conduct extensive research, we need more insight into this regulatory mechanism controlling heat stress responses and tolerance.

### 4.1. Ethylene and Related Post-Translational Modifications

Ethylene stands out as a potent abiotic stress regulator, operating over a wide range of concentrations and inducing multiple changes in plants to mitigate the damage caused by stress [51]. Heat stress has been shown to affect ethylene production mainly in reproductive tissues, such as floral, pedicel, and fruit tissues, and to optimize the dynamics of resource allocation [173]. Exogenous application of ethylene triggers activation of various defense proteins, which are important for maintaining homeostasis in plant cells and promoting thermotolerance [174], and also stimulates various signaling pathways involved in defense against heat stress [107].

Heat stress proteins also fill essential roles in combating high temperature stress, acting to maintain proper cell functioning, growth, and development [9]. They are associated with other defense responses such as protein refolding [175], prevention of protein denaturation and aggregation, membrane stabilization, and induction of antioxidant enzymes, which serve to further preserve cell homeostasis [176]. Studies to date support a direct link between ethylene and rapid synthesis of HSPs at both transcriptional and protein levels [177]. Early evidence also suggests that ethylene induces the accumulation of HSP70 [178] and HSP90 [179], two chaperones that regulate folding and whose cochaperones are associated with signaling, protein targeting, and protein denaturation [180]. Moreover, ACC-like oxidase, which is an important ethylene biosynthesis gene, is the target site for miR5175. According to the studies, ACC-like oxidase mRNA is downregulated in 24 h heat-stressed plants [181,182]. An omics-based approach has shown post-transcriptional regulation of fruit ripening (via miR164-*NAC*). NAC transcription factors are crucial for fleshy fruit ripening [183]. miR164 acts as an upstream regulator of *NAC* transcription factors, and the high abundance of miR164 might abolish the effects of *NAC* transcription factors on fruit ripening. Gene expression analysis and luciferase reporter assays indicated that Ade-miR164 and one of its precursor miRNAs (*Ade-MIR164b*) were repressed by ethylene treatment and negatively correlated with *AdNAC6/7* expression [184]. Thus, considerable evidence has demonstrated that ethylene mediates transcriptional-level regulation; however, research on its contribution to post-translational regulation under heat stress is lacking.

### 4.2. H_2_S and Related Post-Translational Modifications

H_2_S is a lipophilic gaseous molecule that has been revealed as a potent gasotransmitter involved in signal transduction [185]. At low concentration, it can enhance the heat tolerance response in wheat seedlings and positively regulate their growth and development; it was also found to increase total sugar content and CAT, SOD, and APX activity while reducing MDA and ROS [123]. Similarly, exogenous application of H_2_S to maize in the context of heat stress decreased oxidative damage, reduced electrolyte leakage, and improved thermotolerance by modulating the antioxidant system (APX, CAT, SOD, glutathione peroxidase (GPX), glutathione reductase (GR), monodehydroxy ascorbate reductase (MDHAR), ascorbic acid, glutathione, and flavonoids); upregulating gene expression; and increasing soluble sugar, trehalose, and osmolyte (proline, glycine betaine) contents [186].

Post-translational modification of proteins by H_2_S involves the oxidation of cysteine residues to form persulfides [187]. For example, in *Arabidopsis*, persulfidation of Cys160 in the cytosolic GapC1 and GapC2 isoforms of five glyceraldehyde-3-phosphate dehydrogenases can affect either enzyme activity or cytosolic/nuclear partitioning [188,189]. Studies of stress physiology support a strong conclusion that various PTMs such as S-nitrosylation (SNO), S-glutathionylation (SSG), and S-sulfenylation (SOH) may be applied to Cys thiols to improve plant defense responses. These oxidized forms can be reduced by intracellular reducing agents such as GSH, thioredoxin (Trx), and glutaredoxin (Grx) [190]. Under prolonged stress, irreversible modification of thiols occurs, such as with sulfinic (RSO_2_H) and sulfonic acids (RSO_3_H). Persulfidated proteins also have a protective function against the accumulation of ROS/RNS, reacting with the latter to form an adduct (RSSO_3_H) that may be restored by thioredoxin to free thiol [191,192]. Antioxidant enzymes are also subject to PTMs under stress conditions; for example, APX1 is inactivated by oxidation of Cys32, while glutathionylation protects it from irreversible oxidation [193]. The same Cys32 position can also be S-nitrosylated by NO and persulfidated by hydrogen sulfide; these modifications increase the enzyme’s activity [188,194]. PTMs can also have negative feedback effects; in tomato plants under osmotic stress, ethylene regulates stomatal closure and triggers H_2_S production in guard cells, which in turn persulfidates Cys60 of 1-aminocyclopropane-1-carboxylic acid oxidase (ACO1), inhibiting its enzymatic activity and retarding ethylene biosynthesis [195].

However, the direct interaction between miRNAs and H_2_S is documented in recent therapeutic research. miRNAs and H_2_S influence the biosynthesis and expression of each other. As per the evidence, H_2_S released by NaHS and Na_2_S can upregulate miR-133a levels in cultured cardiomyocytes in vitro and exhibits cardioprotective effects in cardiomyocyte hypertrophy [196,197]. Moreover, miR-21 targets SP1 to decrease CSE transcription and H_2_S production [198]. 

## 5. Crosstalk between Ethylene and H_2_S for Heat Stress Tolerance through the Involvement of Sulfur

**Figure 2:**
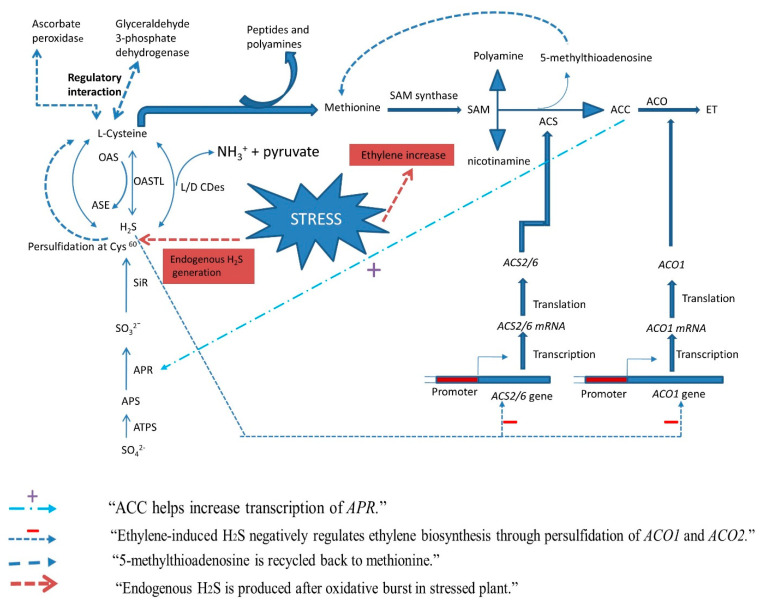
Schematic representation of the regulatory interaction between ethylene and H_2_S biosynthesis for stress tolerance through the S-assimilation pathway. Different arrow types shown above indicate different possible mechanisms. ACC, 1-aminocyclopropane-1-carboxylic acid; ACO, ACC oxidase; ACS, ACC synthase; APR, APS reductase; APS, adenosine 5-phosphosulfate; ATPS, ATP-sulfurylase; Cys, cysteine; DCD, D-cysteine desulfhydrase; LCD, L-cysteine desulfhydrase; ET, ethylene; H_2_S, hydrogen sulfide; NH_3_, ammonia; S, sulfur; SAM, S-adenosyl methionine; SO_4_^2^^−^, sulfate; SO_3_^2−^, sulfite; S^2−^, sulfide; SiR, sulfide reductase; OASTL, O-acetylserine (thiol)-lyase.

Sulfate availability enhances phytohormone-mediated action [79] and is ultimately used to produce Cys, which not only acts as the storage and transport form of reduced S but is required for GSH synthesis and helps in reducing oxidative stress by detoxifying ROS, thereby maintaining the redox state and defense processes required for thermotolerance [199,200]. As the immediate substrate for Cys synthesis, sulfide can be used to synthesize proteins and other organic compounds [201,202,203] that promote heat tolerance [86]; conversely, sulfide can be produced through the degradation of cysteine by desulfhydrases (DESs) [204,205,206]. Sulfide is also an important source of reactive sulfur species (RSS), SAM, GSH, and phytochelatins, and it has been reported to interact with ethylene [69]. That the interaction between ethylene and S promotes abiotic stress tolerance has been established by multiple studies [13,207,208,209,210]. In addition, H_2_S and ethylene can both be regulated by S and in turn can regulate S assimilation to influence stress tolerance. When plants are exposed to excess sulfur in the form of SO_2_, sulfate, or Cys, H_2_S is emitted via foliage into the atmosphere [201,211]. Thus, a regulatory interaction exists between the biosynthetic pathways of ethylene and H_2_S that induces signaling for tolerance of stress conditions via multiple mechanisms (Figure 2).

As described above, cysteine formed during S assimilation is involved in the synthesis of Met and proteins, and it also acts as the S^2−^ donor for H_2_S synthesis. In addition, the ethylene generation and S metabolism pathways are interlinked; treatment with an exogenous ethylene precursor has been shown to increase the activity of APS reductase, which is involved in S assimilation [212]. Similarly, ethylene was found to increase ATP-S activity and S uptake in *Brassica* [80] and to induce H_2_S generation through increased activity of *L*-/*D*-cysteine desulfhydrase in Arabidopsis leaves [68]. Thus, both ethylene and H_2_S could be linked with S in inducing heat stress tolerance. Mechanistically, it could be assumed that heat stress causes oxidative stress, which increases S assimilation and leads to enhanced H_2_S and ethylene synthesis, which regulate antioxidants to scavenge ROS and induce tolerance. A regulatory role has been reported for H_2_S in ethylene-mediated stress responses, highlighting the crosstalk between these three pathways; for example, treatment of peach roots with exogenous H_2_S has been shown to increase endogenous H_2_S content, inhibiting ethylene synthesis and reducing the damage from waterlogging stress [213]. More indirect crosstalk is evidenced in the S-mediated regulation of NO via H_2_S to enhance abiotic stress tolerance; it is also known that a regulatory interaction occurs between NO and ethylene in the context of abiotic stress tolerance [78], and H_2_S and NO crosstalk is associated with inhibition of ethylene biosynthesis [214,215]. Another example of NO and ethylene crosstalk was demonstrated in wild-type *Arabidopsis* calluses under salt stress, in which H₂O₂ enhanced ethylene production while ethylene in turn reduced H₂O₂ generation [216]. In the presence of S, H_2_O_2_ has been shown in drought-exposed wheat to potentiate the defense system and alleviate damage to chloroplasts and photosynthesis [217]. These findings together highlight the interconnection between the three pathways.

By inhibiting stress ethylene synthesis, H_2_S can also improve the activity and proline content of roots, reduce oxidative damage, and alleviate lipid peroxidation [218]. In tomato, H_2_S decreases transcript accumulation of ethylene receptor genes (*SlETR5* and *SlETR6*) and associated transcription factors (*SlCRF2* and *SlERF2*) [213], while in the context of Pb stress, it enhances GSH content and nonprotein thiols to scavenge ROS [219]. H_2_S, ethylene, and S also all contribute to the regulation of sugar content, sugars being important osmolytes in regard to heat stress tolerance. All told, the evidence supports the existence of extensive crosstalk between ethylene, H_2_S, and S pathways in relation to heat tolerance.

## 6. Conclusions

Heat stress is a major constraint on crop productivity, and its severity is likely to increase with the ongoing climate change. Plants leverage various mechanisms for adapting to heat stress, including enhancement of antioxidant potential, synthesis of reduced S-compounds, and activation of signaling hormone biosynthetic pathways. Ethylene and H_2_S are key signaling molecules that regulate the growth and development of plants and are involved in acclimation to abiotic stresses; in particular, they contribute substantially to heat stress tolerance by inducing metabolic changes and post-translational modifications. In addition, the processes for their biosynthesis depend on S-adenosyl methionine and sulfide, respectively, and so are directly or indirectly tied to S assimilation. Consequently, crosstalk between ethylene, H_2_S, and S is an important factor in regulating heat stress, and it can potentially be exploited for maximum alleviation of heat and other stresses in crop plants.

## Figures and Tables

**Table 1:** Selected studies on the crucial role of ethylene in heat stress tolerance. ACC, 1-aminocyclopropane carboxylic acid; ETH, ethephon.

S. No.	Plant	Ethylene Source/Concentration	Temperature Range	Response	Reference
1.	*Agrostis stolonifera*	100 µmol L^−1^ ACC	35 °C	Increased activity of ascorbate peroxidase, superoxide dismutase, and catalase and regulated thermotolerance	[110]
2.	*Cynara cardunculus*	30 µmol L^−1^ ETH	30 °C	Improved seed germination, root growth, and seed vigor	[112]
3.	*Lactuca sativa*	10 μM ACC	35 °C	Improved seed germination performance	[111]
4.	*Oryza sativa*	10 μM ACC	45 °C	Decreased oxidative stress, upregulated antioxidant defense system, and reduced ion leakage	[107]
5.	*Solanum lycopersicum*	1 μL L^−1^ ETH	50 °C	Promoted expression of ethylene-induced responsive genes and improved pollen quality	[47]
6.	*Solanum lycopersicum*	1 μL L^−1^ ETH	50 °C	Alleviated oxidative stress and maintained redox homeostasis	[109]
7.	*Oryza sativa*	1.6 mM ETH	40 °C	Stimulated antioxidant defense system, improved carbohydrate metabolism, and increased photosynthetic and growth attributes	[20]

**Table 2:** Selected studies on the crucial role of H_2_S in heat stress tolerance. NAHS, sodium hydrogen sulfide.

S. No.	Plant	H_2_S Source	Temperature Range	Response	References
1.	*Fragaria*	100 µM NAHS	42 °C	Increased activity of antioxidant enzymes and increased expression of antioxidant enzymes	[131]
2.	*Nicotiana tabacum*	50 µM NAHS	42 °C	Increased vitality of cells and alleviated electrolyte leakage	[122]
3.	*Nicotiana tabacum*	50 µM NAHS	43 °C	Increased S-containing compounds such as cysteine and glutathione as well as antioxidant enzymes	[132]
4.	*Zea mays*	1.2 mmol NAHS	47 °C	Decreased oxidative stress and upregulated antioxidant defense system	[133]
5.	*Zea mays*	1.5 mmol NAHS	38 °C	Increased proline biosynthesis	[134]
6.	*Zea mays*	0.5 mmol NAHS	47 °C	Increased betaine accumulation	[135]
7.	*Zea mays*	500 µM NAHS	48 °C	Increased endogenous H_2_S accumulation	[132]

**Table 3:** Selected studies on the crucial role of sulfur in heat stress tolerance.

S. No.	Plant	Sulfur Concentration	Temperature Range	Response	Reference
1.	*Brassica napus*	8.7 μM	33 °C	Improved grain quality and enhanced nutritional compounds	[142]
2.	*Brassica napus*	500 ppm	28 °C	Improved growth, yield, and physiological characteristics	[143]
3.	*Brassica napus*	500 ppm	28 °C	Improved physiological and yield characteristics	[144]
4.	*Cymopsis tetragonoloba*	100 mg S kg^−1^ soil	45 °C	Enhanced carbohydrate metabolism and mitigated oxidative damage	[140]
5.	*Solanum lycopersicum*	2–8 ppm	45 °C	Improved growth, photosynthesis, and biochemical attributes	[145]
6.	*Triticum aestivum*	130 kg ha^−1^ S-coated urea	33 °C	Improved growth rate, yield, physiological parameters, and N content	[146]

## Data Availability

Data presented in this review article are original.
